# Supplementary material for: The Intraflagellar Transport Protein IFT20 Recruits ATG16L1 to Early Endosomes to Promote Autophagosome Formation in T Cells
Source: Front Cell Dev Biol. 2021 Mar 22;9:634003. doi: 10.3389/fcell.2021.634003 (PMC8019791; doi:10.3389/fcell.2021.634003)
Supplement: Supplementary file 1 [file Data_Sheet_1.PDF]

SUPPLEMENTARY FIGURE LEGENDS

Supplementary figure 1. (A) Representative IFT20 immunoblot on lysates from Jurkat cells, transfected with either empty vector (GFP), with the IFT20-GFP construct (IFT20-GFP), or with the plasmid encoding for ΔCC IFT20-GFP (ΔCC IFT20-GFP). Actin was used as a loading control. (B) Immunoblot analysis of IFT20 in lysates of control and IFT20KD Jurkat cells (mean fold ± SD; one-sample t test; $n > 3$). (C) Representative IFT20 immunoblot on lysates from control and IFT20 knocked-down (KD) cells transiently transfected with empty vector (GFP), with the IFT20-GFP construct or with ΔCC IFT20-GFP vector. Actin was used as a loading control. (D) Immunoblot analysis of GMAP210 in lysates of control and GMAP210KD Jurkat cells (mean fold ± SD; one-sample t test; n=3). The migration of molecular mass markers is indicated; #, non-specific signal. *$P < 0.05$; ***$P<0.0001$

Supplementary figure 2. Quantification of Mander's colocalization coefficient (mean ± SD; ≥ 28 cells, n = 3) between BECLIN 1 and GM130 in medial confocal sections of control or IFT20KD Jurkat cells stained with the respective antibodies. Representative images are shown. Size bar: 5 μm.

Supplementary figure 3. Representative immunoblot anti-ATG16L1 (A), anti-Rab5 (B), anti-BECLIN 1 (C) and anti-ATG5 (D) on lysates from control and IFT20KD Jurkat cells. The quantification of the relative protein expression is normalized to control (mean fold ± SD; n=3). The migration of molecular mass markers is indicated.

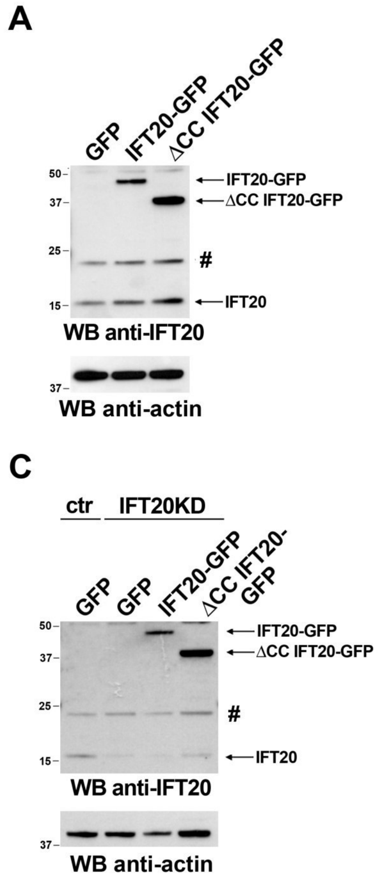

Supplementary figure 1

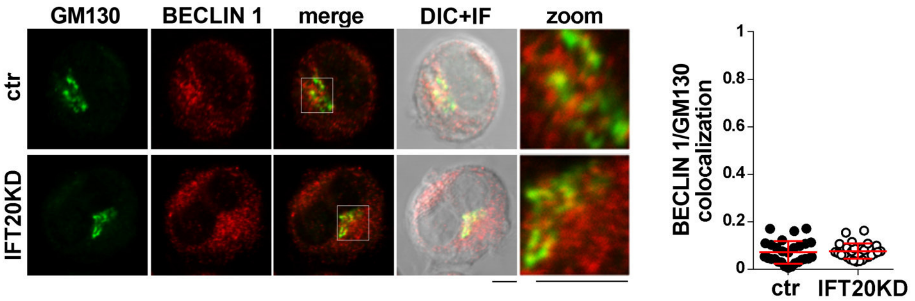

Supplementary figure 2

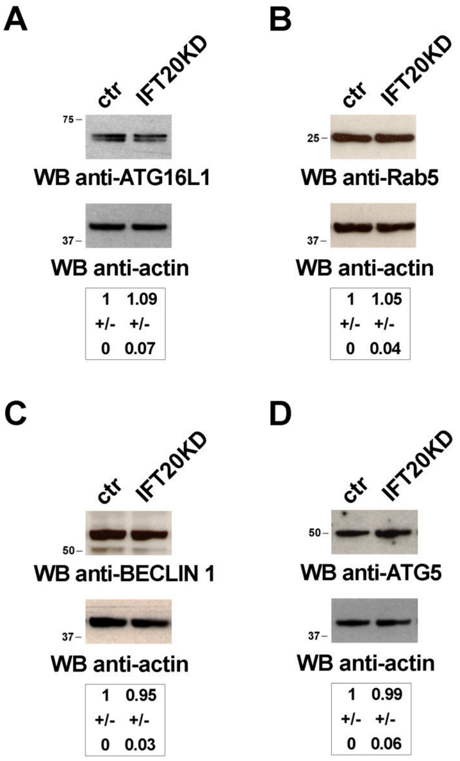

Supplementary figure 3

SUPPLEMENTARY TABLES

Table S1. List of the primers used in this study

Primers	Sequence
**ΔCC mutant-GFP XhoI Fw**	CCGCTCGAGATGGCCAAGGACATCCTG
**ΔCC mutant-GFP KpnI Rev**	CGGGGTACCCCGATGGCCTTCATCTTT
**CC mutant-GFP XhoI Fw**	CCGCTCGAGATGGGTGCTCGGAACT
**CC mutant-GFP KpnI Rev**	CGGGGTACCCCTTTCTGAAAAATAAATTGGTC
**GST-ΔCC mutant EcoRI Fw**	CCGGAATTCCGATGGCCAAGGACAT
**GST-ΔCC mutant XhoI Rev**	CCGCTCGAGTCAGATGGCCTTCATCTTTTC
**GST-CC mutant EcoRI Fw**	CCGGAATTCCGATGGGTGCTCGGAA
**GST-CC mutant XhoI Rev**	CCGCTCGAGTCATTTCTGAAAAATAAATTGGTCAA
**GST-IFT20 EcoRI Fw**	CCGGAATTCCGATGGCCAAGGACAT
**GST-IFT20 XhoI Rev**	CCGCTCGAGTCATTTCTGAAAAATAAATTGGTCAA

Table S2. List of the antibodies used in this study

Antibody	Host Species	Catalogue number	Source	Dilution WB	Dilution IF
Anti-actin	mouse	MAB1501	EMD Millipore	1:10000	-
Anti-ATG5	mouse	sc-133158	Santa Cruz	1:500	-
Anti-Atg16L1	mouse	sc-393274	Santa Cruz	1:500	1:50
Anti-Atg16L1	rabbit	8089S	Cell Signaling	1:500	-
Anti-BECLIN 1	mouse	sc-48341	Santa Cruz	1:500	-
Anti-BECLIN 1	rabbit	3495T	Cell Signaling	1:500	1:50
Anti-ERK2	rabbit	sc-154	Santa Cruz	1:500	-
Anti-γ-tubulin	mouse	T6557	Sigma Aldrich	-	1:200
Anti-giantin	rabbit	ab80864	Abcam	-	1:200
Anti-GFP	rabbit	A11122	Life Technologies	1:1000	1:200
Anti-GMAP210	mouse	611712	BD Biosciences	1:250	-
Anti-GM130	rabbit	610822	BD Biosciences	1:500	1:100
Anti-IFT20	rabbit	-	GJ Pazour*	1:500	1:200
Anti-LC3B	rabbit	3868	Cell Signaling	1:500	1:200
Anti-Rab5	mouse	610724	BD	1:2000	1:50
Anti-Rab5	rabbit	3547S	Cell Signaling	-	1:200
Anti-pericentrin	rabbit	Ab4448	Abcam	-	1:300

*Program in Molecular Medicine, University of Massachusetts Medical School, Worcester, MA 01605, USA.
